# Beyond STRs: Integrative Forensic Genomics from MPS to Genetic Genealogy and AI-Based Prediction

**DOI:** 10.3390/genes17050580

**Published:** 2026-05-18

**Authors:** Desiree Brancato, Elvira Coniglio, Francesca Bruno, Simone Treccarichi, Mirella Vinci, Francesco Calì, Salvatore Saccone, Concetta Federico

**Affiliations:** 1 Department Biological, Geological and Environmental Sciences, University of Catania, 95124 Catania, Italy; desiree.brancato@phd.unict.it (D.B.); elvira.coniglio@phd.unict.it (E.C.); or federico@unict.it (C.F.); 2 Department Medicine and Surgery, Kore University of Enna, 94100 Enna, Italy; francesca.bruno@unikore.it (F.B.); cali@oasi.en.it (F.C.); 3 Oasi Research Institute-IRCCS, 94018 Troina, Italy; streccarichi@oasi.en.it (S.T.); mvinci@oasi.en.it (M.V.)

**Keywords:** forensic genomics, massively parallel sequencing (MPS), single nucleotide polymorphisms (SNPs), microhaplotypes, forensic genetic genealogy (FGG), DNA phenotyping, artificial intelligence, forensic intelligence

## Abstract

Recent advances in forensic genetics are rapidly transforming the field from traditional DNA profiling toward integrative and predictive genomic approaches. While short tandem repeat (STR)-based typing remains the gold standard for human identification, emerging technologies such as massively parallel sequencing (MPS), forensic genetic genealogy (FGG), and artificial intelligence (AI)-driven bioinformatics are expanding the scope of forensic investigations, with MPS also widely established in clinical genomics, further supporting its application in complex and unresolved cases. This article presents a structured narrative and conceptual review of next-generation forensic genomics, based on selected peer-reviewed studies, technical guidelines, and recent review articles relevant to MPS-based marker analysis, FGG, DNA phenotyping, ancestry inference, AI-supported bioinformatics, validation, and ethical/legal issues. We discuss the transition from STRs to single nucleotide polymorphisms (SNPs) and microhaplotypes enabled by MPS, emphasizing their applications in mixture deconvolution, kinship analysis, and degraded DNA samples. The role of FGG in cold case resolution is examined, alongside methodological, legal, and ethical considerations related to the use of public genetic databases. Furthermore, we explore recent developments in DNA phenotyping and ancestry inference, focusing on predictive models of externally visible characteristics (EVCs) and their forensic utility. Particular attention is given to the growing impact of AI and machine learning in data interpretation, probabilistic genotyping, and pattern recognition across complex genomic datasets. Finally, we address current limitations, including technical standardization, population biases, data privacy concerns, and the need for robust validation frameworks. Rather than providing a systematic review, this work aims to synthesize current developments into an operational framework for integrated forensic genomics, distinguishing forensic intelligence, probabilistic interpretation, confirmatory testing, and evidentiary use. By integrating technological, analytical, and ethical perspectives, this review proposes a conceptual framework for integrated forensic genomics, in which genomic data are used not only for identification but also for forensic intelligence generation.

## 1. Introduction

### 1.1. Scope of Integrated Forensic Genomics

Forensic genetics has undergone a profound transformation over the past three decades, evolving from a discipline focused on short tandem repeat (STR) profiling to a multifaceted field increasingly driven by genomic technologies and computational analytics. Traditional STR-based DNA typing has long represented the gold standard for human identification due to its robustness, high discriminatory power, and compatibility with established forensic databases [1,2]. However, despite its success, this approach presents intrinsic limitations when applied to complex forensic scenarios, including degraded samples, low-template DNA, and complex DNA mixtures [3,4].

The advent of massively parallel sequencing (MPS), also referred to as next-generation sequencing (NGS), has significantly expanded the scope of forensic genetic analysis. By enabling the simultaneous interrogation of thousands of loci, MPS facilitates the analysis of single-nucleotide polymorphisms (SNPs), microhaplotypes, and complete mitochondrial genomes, thereby providing enhanced resolution in challenging cases [5,6]. These advances have opened new avenues for addressing previously intractable forensic questions, particularly in the context of degraded biological material and distant kinship inference.

In parallel, the emergence of forensic genetic genealogy (FGG) has introduced a paradigm shift in investigative practices. By leveraging large-scale genomic databases and genealogical inference, FGG has demonstrated remarkable success in resolving cold cases and identifying unknown individuals, thereby extending the utility of genetic data beyond direct matching to database profiles [7]. Similarly, the development of forensic DNA phenotyping (FDP) approaches has enabled the probabilistic prediction of externally visible characteristics (EVCs), such as eye, hair, and skin color, as well as biogeographical ancestry, providing investigative leads in the absence of reference profiles [8].

More recently, the integration of artificial intelligence (AI) and advanced bioinformatics tools has further transformed forensic genomics. Machine learning algorithms are increasingly applied to mixture deconvolution, probabilistic genotyping, and pattern recognition in high-dimensional genomic datasets, enhancing both analytical sensitivity and interpretative accuracy [9,10]. These developments are contributing to the emergence of a new conceptual framework in which forensic genetics operates not only as an identificatory tool but also as a source of forensic intelligence.

This review aims to provide an integrative perspective on these converging advancements, highlighting how MPS, forensic genetic genealogy, DNA phenotyping, and AI-driven bioinformatics collectively redefine the boundaries of forensic genetics. By moving beyond a technology-centric description toward a systems-level understanding, we propose a framework for “integrated forensic genomics” in which multiple genomic and computational approaches contribute to distinct but interconnected layers of forensic interpretation, including investigative lead generation, probabilistic inference, confirmatory testing, court-admissible genetic evidence, and AI-assisted decision-support systems. Within this framework, different data types are not considered equivalent in evidential value but are integrated according to their analytical robustness, validation status, interpretative uncertainty, and forensic applicability. This approach reflects the ongoing transition from traditional identification toward predictive, intelligence-driven, and data-integrative forensic science.

### 1.2. Literature Scope and Review Approach

This article was conceived as a structured narrative and conceptual review aimed at integrating major developments in forensic genomics within a unified analytical framework. The literature considered for this review was selected based on relevance to the main themes addressed in the manuscript, including MPS, FGG, FDP, ancestry inference, probabilistic genotyping, AI-supported bioinformatics, validation, and ethical/legal implications.

Peer-reviewed articles, technical guidelines, seminal studies, and recent review papers indexed in PubMed, Scopus, and Web of Science were preferentially considered. Given the rapidly expanding nature of the field, this work was not intended to be fully systematic or exhaustive; rather, representative and methodologically relevant studies were selected to provide a balanced and integrative overview while maintaining readability and conceptual coherence. Accordingly, the manuscript prioritizes critical synthesis and conceptual integration over exhaustive bibliographic coverage.

## 2. From STRs to MPS-Based Forensic Genomics

**Figure 1:**
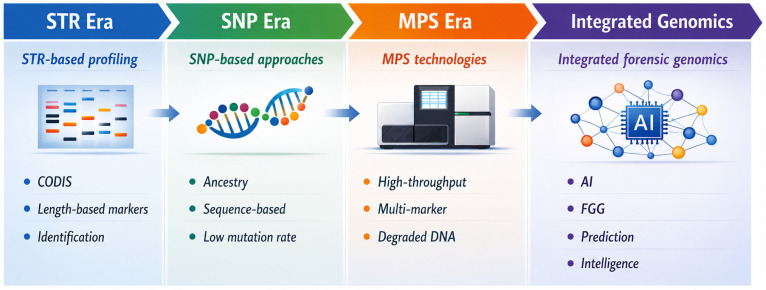
Evolution of forensic genomics. Schematic timeline illustrating the evolution of forensic genetics from traditional STR-based profiling to integrated genomic approaches. The STR era is characterized by length-based markers and database-driven identification (e.g., Combined DNA Index System, CODIS). The subsequent SNP era introduced sequence-based variation, enabling applications such as ancestry inference and DNA phenotyping with improved performance on degraded samples. The advent of MPS marked a transition to high-throughput, multi-marker analysis, significantly enhancing resolution and analytical flexibility. The final stage, integrated forensic genomics, represents the convergence of MPS, FGG, predictive modeling, and artificial intelligence, enabling a shift from simple identification toward predictive and intelligence-driven forensic science.

The evolution of forensic genetics from traditional STR-based profiling to modern integrative genomic approaches reflects a progressive expansion in both analytical resolution and investigative capabilities (Figure 1). The foundation of modern forensic genetics has long relied on STR analysis, a methodology that combines high discriminatory power with standardized protocols and well-established reference databases. STR typing has proven highly effective for individual identification and kinship analysis, particularly in cases involving high-quality DNA samples [1]. However, several intrinsic limitations of STR-based approaches have become increasingly evident, particularly in the context of degraded DNA, low-template samples, and complex DNA mixtures [3].

Over the past decade, MPS technologies have emerged as a transformative platform in forensic genomics, enabling the simultaneous analysis of a wide spectrum of genetic markers, including SNPs, microhaplotypes, insertion–deletion polymorphisms (InDels), and complete mitochondrial genomes [5,11]. Unlike capillary electrophoresis-based STR typing, MPS provides sequence-level data, allowing the detection of intra-repeat variation and increasing the resolution of genetic profiles [12].

One of the most significant advantages of MPS lies in its ability to analyze highly fragmented DNA, a common feature of forensic samples recovered from crime scenes, disaster victim identification (DVI) contexts, and historical remains. In particular, SNPs are well suited for degraded DNA due to their short amplicon size requirements, enabling robust genotyping even in challenging conditions [13]. Furthermore, microhaplotypes—clusters of closely linked SNPs within small genomic regions—have demonstrated promising performance in several forensic applications, particularly in mixture deconvolution and ancestry inference, offering a promising alternative to traditional STR markers [14]. A comparative overview of the main characteristics, forensic applications, strengths, and limitations of these marker systems is summarized in Table 1.

The transition from STRs to MPS-based approaches reflects a broader conceptual shift from targeted profiling toward genome-informed and data-rich forensic analysis. As highlighted in recent integrative reviews of emerging forensic technologies, including those focusing on next-generation sequencing, forensic genetic genealogy, DNA phenotyping, and advanced analytical frameworks, the field is progressively moving toward multi-marker and multi-dimensional datasets that require sophisticated bioinformatic pipelines for interpretation [5,7,8,9,15]. However, many existing reviews primarily focus on specific technological domains or individual applications, with comparatively less emphasis on their integration within unified forensic workflows and intelligence-oriented analytical frameworks.

Importantly, the adoption of MPS in forensic science parallels its widespread implementation in clinical genomics, where standardized sequencing workflows, quality-control procedures, and bioinformatic pipelines have undergone extensive optimization and harmonization. The ability to simultaneously interrogate large portions of the genome through targeted panels, whole-exome sequencing (WES), or whole-genome sequencing (WGS) has substantially expanded genomic analytical capabilities across multiple biomedical fields. Moreover, advances in bioinformatic pipelines and variant interpretation frameworks, increasingly supported by machine learning approaches, have improved the reproducibility and analytical robustness of genomic findings.

At the same time, forensic genomics presents distinct analytical and operational challenges, including low-template and degraded DNA, stochastic effects, contamination control, mixture interpretation, chain-of-custody requirements, and the statistical evaluation of evidence through likelihood-ratio frameworks. In this context, the methodological convergence between clinical and forensic genomics lies not in their applications, but in the increasing need for standardized sequencing workflows, robust bioinformatic pipelines, quality-control metrics, and reproducible interpretation frameworks adapted to domain-specific requirements. Together, these considerations underscore the potential for cross-disciplinary innovation in sequencing workflows, analytical standardization, and genomic data interpretation across forensic and clinical settings.

Despite these advances, the implementation of MPS in routine forensic practice remains heterogeneous, with challenges related to validation, standardization, cost, and legal admissibility. In forensic genomics, validation requires rigorous assessment of analytical sensitivity, specificity, reproducibility, contamination control, sequencing error profiles, allele balance, stochastic thresholds, and drop-in/drop-out behavior, particularly when dealing with low-template or degraded DNA samples and complex mixtures. Additional challenges include the representativeness of population reference databases, harmonization of nomenclature and reporting standards, and the statistical interpretation of evidence through likelihood-ratio frameworks [4,16,17]. Nonetheless, its potential to enhance resolution, improve mixture interpretation, and enable the analysis of novel marker systems positions MPS as a cornerstone of next-generation forensic genomics.

## 3. Emerging Genetic and Epigenetic Markers in Forensic Genomics

The transition toward MPS-based forensic genomics has enabled the development and implementation of a wide range of novel genetic and epigenetic markers that extend far beyond traditional STR profiling. These emerging markers—primarily SNPs, microhaplotypes, InDels, mitochondrial DNA (mtDNA) sequences, and epigenetic signatures—are redefining the analytical capabilities of forensic genetics, particularly in challenging scenarios involving degraded DNA, complex mixtures, and the need for predictive inference [18].

### 3.1. Single Nucleotide Polymorphisms (SNPs)

SNPs represent the most abundant type of genetic variation in the human genome and have become central to next-generation forensic applications. Their low mutation rate and short amplicon requirements make them particularly suitable for degraded DNA samples [19,20]. SNP panels have been developed for multiple forensic purposes, including individual identification, ancestry inference, and DNA phenotyping.

In the context of FDP, SNPs located in genes associated with pigmentation traits have been extensively investigated. Variants within loci such as *HERC2* and *OCA2* are strongly associated with eye color variation, with specific polymorphisms (e.g., rs12913832) playing a major regulatory role [8]. Notably, recent work has demonstrated that the chromatin organization surrounding key pigmentation-associated SNPs is evolutionarily conserved, supporting the functional relevance of these regulatory regions while also highlighting the complexity of genotype–phenotype relationships across different biological and population contexts [21]. Furthermore, comprehensive reviews of forensic DNA phenotyping have highlighted the growing accuracy of SNP-based predictive models for EVCs, while also emphasizing their limitations and population-specific biases [22].

### 3.2. Microhaplotypes

Microhaplotypes are defined as short genomic regions (typically <200 bp) containing two or more closely linked SNPs that can be phased into haplotypes. These markers combine the advantages of SNPs (low mutation rate, short amplicons) with a higher polymorphic information content due to haplotype diversity [14].

Microhaplotypes have demonstrated promising and, in some contexts, improved performance in mixture deconvolution compared to both STRs and individual SNPs, particularly in cases involving multiple contributors and complex DNA mixtures. However, their analytical performance may vary depending on factors such as panel design, sequencing depth, contributor ratios, and population-specific haplotype frequencies. Their ability to provide phase information without requiring long sequencing reads makes them especially valuable in forensic contexts [23]. In addition, microhaplotypes are increasingly being explored for ancestry inference and kinship analysis, with growing evidence supporting their informativeness across diverse populations [24].

### 3.3. Insertion–Deletion Polymorphisms (InDels)

InDels represent another type of biallelic markers that combine some of the advantages of STRs and SNPs. Their length-based variation allows for detection via capillary electrophoresis, while their low mutation rate and short amplicon size make them suitable for degraded DNA [25]. Although less informative than STRs or microhaplotypes in terms of discriminatory power, InDels are often included in multiplex panels to complement other marker systems.

### 3.4. Mitochondrial DNA and Whole Mitogenome Sequencing

Mitochondrial DNA (mtDNA) analysis has long been employed in forensic genetics due to its high copy number per cell, which enhances its recoverability from degraded samples such as hair shafts and skeletal remains [26]. The advent of MPS has enabled full mitochondrial genome sequencing, significantly increasing discriminatory power compared to traditional control region analysis [26,27,28].

However, mtDNA analysis presents limitations, including maternal inheritance and lower individual specificity. Despite these constraints, it remains a critical tool in missing persons identification and historical investigations.

### 3.5. Epigenetic Markers

Epigenetic markers, particularly DNA methylation patterns, have emerged as promising tools for forensic inference. Unlike sequence-based genetic variants, epigenetic markers are dynamic molecular signatures influenced by tissue type, aging, and environmental factors. Consequently, epigenetic modifications can provide information about dynamic biological traits such as age, tissue type, and environmental exposure [29].

DNA methylation-based age prediction models have achieved relatively high predictive accuracy and are increasingly being considered for forensic applications [30,31]. Additionally, tissue-specific methylation signatures can assist in identifying the biological origin of forensic samples (e.g., blood, saliva, semen), thereby providing contextual information that complements genetic profiling.

A comparative overview of the main classes of emerging genetic markers, including their key features, applications, strengths, and limitations, is provided in Table 2.

### 3.6. Toward Multi-Layered Forensic Genomics

The integration of these diverse marker systems reflects a broader shift toward multi-layered forensic genomics, where different types of genetic and epigenetic information are combined to maximize investigative potential. Importantly, while individual marker systems have been extensively reviewed, the challenge—and opportunity—lies in their integration within unified analytical frameworks. Such integration is essential for fully leveraging the capabilities of MPS and advanced bioinformatics in modern forensic investigations and for enabling multi-dimensional forensic inference.

Within this perspective, integrated forensic genomics may be viewed as a modular and hierarchical analytical framework in which different genomic and computational components contribute distinct categories of forensic information. Depending on their level of validation, analytical robustness, and forensic applicability, these components may support investigative lead generation, probabilistic inference, confirmatory testing, or AI-assisted decision-support processes. Importantly, the various layers of information are not intended to carry equivalent evidential weight, but rather to provide complementary and context-dependent contributions within structured forensic workflows.

## 4. Forensic Genetic Genealogy (FGG)

FGG represents one of the most transformative developments in modern forensic science, bridging the gap between traditional DNA profiling and large-scale population genomics. Unlike conventional forensic approaches, which rely on direct matches between crime scene DNA profiles and reference databases (e.g., CODIS), FGG leverages high-density SNP data and genealogical inference to identify individuals through their relatives [7]. This shift from direct matching to kinship-based inference substantially expands the investigative scope of forensic genetics, particularly in cases where no exact database match is available. A direct comparison between traditional STR-based DNA profiling and forensic genetic genealogy is provided in Table 3.

### 4.1. Principles and Workflow of FGG

FGG operates through a multi-step process that combines genomic data with genealogical research. Typically, DNA extracted from a forensic sample is genotyped using SNP arrays or sequenced using MPS-based SNP panels, generating a dense genomic profile compatible with direct-to-consumer (DTC) genetic databases. The resulting profile is then uploaded—under specific legal frameworks—to public or law-enforcement-accessible genealogy databases such as GEDmatch or FamilyTreeDNA [32].

The workflow involves three main steps:

- *Database matching*—Identification of individuals sharing significant segments of DNA (identity-by-descent, IBD) with the unknown sample.
- *Genealogical reconstruction*—Building family trees based on shared matches, historical records, and demographic data.
- *Candidate narrowing*—Integration of genetic, geographical, and contextual information to identify potential suspects or unknown individuals.

This approach allows investigators to identify distant relatives (e.g., third or fourth cousins), significantly expanding the investigative reach beyond traditional forensic databases.

### 4.2. Applications in Criminal Investigations and Cold Cases

FGG has gained widespread attention in recent years following its successful application in high-profile cases, most notably the identification of the “Golden State Killer” in 2018. Since then, genealogical approaches have contributed to the resolution of numerous cold cases and the identification of previously unknown individuals, particularly in the United States, where forensic genetic genealogy has been most extensively implemented [7,33]. Recent reviews and published case compilations indicate that several hundred investigations in the U.S. have benefited from FGG-based approaches, although adoption and legal permissibility vary substantially across jurisdictions and countries [34,35,36].

FGG is particularly valuable in cases where:

- no direct database match is available
- DNA samples are degraded but still suitable for SNP profiling
- long-term unidentified remains require kinship-based identification

In DVI and missing persons investigations, FGG can be used as a complementary tool to traditional kinship analysis, extending the search to more distant relatives, thereby increasing the likelihood of identification.

### 4.3. Technical Considerations

The success of FGG depends on several technical factors, including:

- the density and quality of SNP data
- compatibility with genealogy databases
- accurate detection of IBD segments
- robust bioinformatic pipelines for kinship inference

MPS technologies play a key role in enabling FGG, particularly when working with degraded forensic samples. Compared to traditional microarray-based genotyping, MPS offers greater flexibility in marker selection and improved analytical performance in challenging conditions [5].

However, differences between forensic SNP panels and DTC genotyping arrays can complicate data compatibility, necessitating imputation strategies or customized bioinformatic pipelines. In forensic contexts, imputation procedures may introduce additional uncertainty, particularly when applied to degraded or low-coverage DNA samples characterized by missing loci or incomplete genomic profiles. Moreover, imputation accuracy may vary across populations depending on the representativeness of available reference datasets, with reduced performance often reported for underrepresented ancestries. Consequently, the use of imputed data in forensic genetic genealogy requires careful validation, transparent reporting of uncertainty, and appropriate interpretation within probabilistic frameworks.

### 4.4. Limitations and Biases

Despite its remarkable success, FGG presents several limitations:

- *Database bias*: most genealogy databases are heavily skewed toward individuals of European ancestry, reducing effectiveness for underrepresented populations [32].
- *False leads*: distant genetic matches can generate complex genealogical networks, increasing the risk of misinterpretation.
- *Data quality constraints*: low-coverage or degraded DNA may limit the accuracy of kinship inference.

Moreover, the probabilistic nature of genealogical reconstruction requires careful interpretation and corroboration with non-genetic evidence.

### 4.5. Ethical and Legal Considerations

FGG raises significant ethical and legal questions, particularly concerning genetic privacy, informed consent, and the use of consumer genetic data for law enforcement purposes. The use of publicly accessible genealogy databases has sparked significant debate over whether individuals who upload their genetic data for recreational purposes fully understand the potential forensic implications [33,37].

Regulatory frameworks vary widely across jurisdictions. In the United States, law enforcement use of FGG is subject to evolving guidelines and agency-specific policies, while in Europe, the application of forensic genetic genealogy is influenced by complex data protection frameworks, including the General Data Protection Regulation (GDPR), national legislation, law-enforcement directives, proportionality requirements, and procedural safeguards governing the processing of sensitive genetic data.

### 4.6. FGG Within the Framework of Integrated Forensic Genomics

FGG exemplifies the transition from traditional identification toward forensic intelligence in forensic science. By linking genomic data with genealogical and demographic information, FGG expands the interpretative scope of forensic genetics toward multi-dimensional inference.

Importantly, FGG does not operate in isolation but is increasingly integrated with other emerging approaches, including DNA phenotyping and AI-driven analytics. This convergence enables a multi-dimensional reconstruction of unknown individuals, combining genetic relationships, predicted physical traits, and probabilistic inference models.

As such, FGG represents a central component of the emerging paradigm of integrated forensic genomics, in which diverse data sources are combined to generate actionable investigative leads in complex forensic scenarios.

## 5. DNA Phenotyping and Ancestry Inference

FDP and biogeographical ancestry inference represent two of the most innovative applications of forensic genomics, extending the use of genetic data beyond individual identification toward the prediction of EVCs and population origin. These approaches provide valuable investigative leads in cases where no reference DNA profile is available, thereby complementing traditional forensic methodologies [8].

### 5.1. Principles of Forensic DNA Phenotyping

FDP relies primarily on the analysis of SNPs associated with phenotypic traits, including eye color, hair color, skin pigmentation, and, more recently, facial morphology. These SNPs are typically located within genes involved in pigmentation pathways, such as *HERC2*, *OCA2*, *SLC24A5*, and *MC1R*, which influence melanin production and distribution [8].

Among these, the SNP rs12913832 within the *HERC2/OCA2* regulatory region is one of the most extensively studied variants, showing a strong association with blue versus brown eye color. Functional and evolutionary analyses have demonstrated that the chromatin organization surrounding this locus is highly conserved among vertebrates, supporting its biological relevance in pigmentation pathways, although predictive performance in forensic contexts remains dependent on population structure, marker panels, and analytical models [21].

Recent advances have improved the predictive accuracy of FDP models through the integration of multiple SNPs into probabilistic frameworks. Systems such as HIrisPlex and HIrisPlex-S enable simultaneous prediction of eye, hair, and skin color with relatively high accuracy across diverse populations [38,39]. Nevertheless, predictive performance remains variable depending on population structure and the complexity of the trait.

### 5.2. Genetic Architecture of Phenotypic Traits

The genetic basis of EVCs ranges from relatively simple architectures (e.g., eye color) to highly polygenic traits (e.g., facial morphology and height). While a limited number of SNPs can explain a significant proportion of eye and hair color variation, more complex traits require the integration of hundreds or thousands of variants, often with small individual effect sizes. Predictive performance in forensic DNA phenotyping is therefore highly variable across traits and populations and is commonly evaluated using statistical metrics such as area under the curve (AUC), sensitivity, specificity, and predictive accuracy. In general, pigmentation traits such as eye and hair color show substantially higher predictive performance than highly polygenic traits such as facial morphology or height.

Importantly, these traits differ substantially in biological complexity, forensic maturity, validation status, and practical applicability. Pigmentation traits such as eye, hair, and skin color are currently the most robust and widely implemented FDP applications, whereas facial morphology and highly polygenic traits remain substantially more challenging and less standardized in forensic contexts. Moreover, epigenetic age estimation represents a distinct category of inference based on dynamic molecular signatures rather than externally visible characteristics.

From a broader genomic perspective, the relationship between genotype and phenotype is influenced not only by sequence variation but also by regulatory complexity and population-specific genetic architecture. This complexity underscores the need for integrative probabilistic models that go beyond simple genotype–phenotype associations. A comparative overview of major phenotypic traits, their genetic architecture, representative predictive metrics, and forensic utility is presented in Table 4.

### 5.3. Biogeographical Ancestry Inference

Ancestry inference is based on the analysis of ancestry-informative markers (AIMs), typically SNPs that exhibit substantial allele frequency differences among populations from different geographical regions. These markers allow the estimation of an individual’s genetic ancestry at continental or sub-continental levels [40,41,42].

Ancestry inference can provide valuable investigative information, particularly in the absence of database matches. However, it is important to distinguish between genetic ancestry and socially defined concepts of ethnicity, as the two are not equivalent and may lead to misinterpretation if not carefully communicated.

### 5.4. Predictive Models and Machine Learning Approaches

Recent developments in FDP have increasingly incorporated machine learning and statistical modeling approaches to improve predictive performance. These models integrate multiple genetic variants and, in some cases, environmental and demographic data to generate probabilistic predictions of phenotypic traits.

Machine learning approaches are particularly relevant for complex traits, where traditional regression-based models may be insufficient. However, the interpretability of these models and their robustness across populations remain critical challenges. In forensic applications, the reliability and admissibility of machine-learning-based predictions depend on rigorous validation procedures, including appropriate separation of training and test datasets, external validation, cross-validation strategies, calibration of predicted probabilities, and transparent reporting of uncertainty. Additional concerns include overfitting, class imbalance, population stratification, and the limited explainability of complex predictive models, particularly in contexts where probabilistic outputs may influence investigative or judicial decision-making.

### 5.5. Limitations and Challenges

Despite significant progress, FDP and ancestry inference face several limitations:

- *Population bias*: most predictive models are trained on datasets of predominantly European ancestry, reducing accuracy in other populations.
- *Trait complexity*: many phenotypes are polygenic and influenced by environmental factors.
- *Probabilistic nature*: predictions are inherently probabilistic and should not be interpreted as definitive identifications.
- *Ethical concerns*: the use of FDP raises issues related to privacy, potential stigmatization, and the risk of reinforcing racial profiling.

These limitations highlight the importance of careful validation, transparent reporting, and responsible use of predictive genetic information in forensic contexts.

### 5.6. FDP Within Integrated Forensic Genomics

Within the framework of integrated forensic genomics, FDP and ancestry inference serve as key components of forensic intelligence. When combined with FGG and MPS-based marker analysis, these approaches contribute to a multi-dimensional reconstruction of unknown individuals.

Rather than replacing traditional identification methods, FDP complements them by providing additional layers of information that can guide investigations. This integration reflects a broader shift toward predictive and data-driven forensic science, where genetic information is used not only to identify individuals but also to infer biological and demographic characteristics.

## 6. AI and Bioinformatics in Forensic Genomics

The rapid expansion of forensic genomics, driven by massively parallel sequencing (MPS) and multi-marker approaches, has generated increasingly complex and high-dimensional datasets. This transformation has required the development of advanced bioinformatic pipelines and the integration of AI methodologies to enable accurate, reproducible, and scalable data interpretation. As a result, bioinformatics and AI are now central components of modern forensic genomics, bridging the gap between raw genomic data and actionable forensic intelligence.

### 6.1. Bioinformatic Pipelines in Forensic Genomics

**Figure 2:**
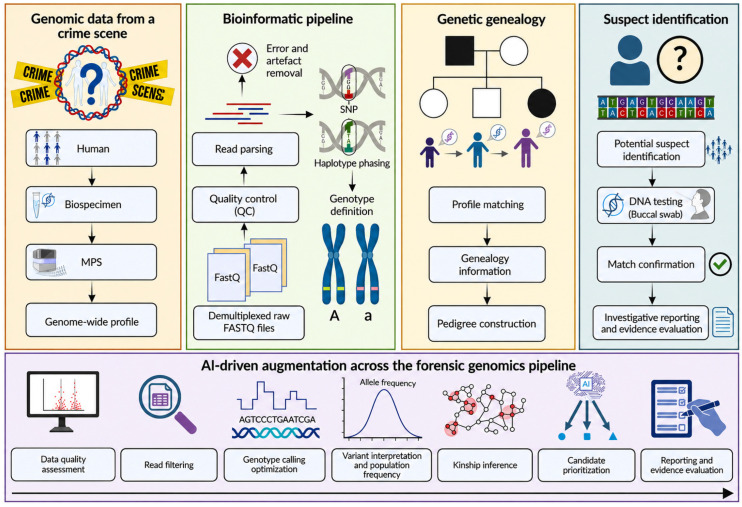
AI-augmented forensic genomics pipeline from raw MPS data to investigative lead identification. Genomic material recovered from a crime scene undergoes DNA extraction and MPS, generating genome-wide data (e.g., SNP profiles). Raw sequencing data (FASTQ files) are processed through a bioinformatic pipeline including quality control, read alignment, variant calling, and genotype definition. The resulting genetic profile can be leveraged for FGG, where profile matching against public or forensic databases enables the identification of putative relatives, followed by pedigree reconstruction and kinship inference. This process supports the prioritization of potential investigative leads, which are subsequently validated through direct DNA testing and statistical evaluation (e.g., likelihood ratios). AI and machine learning (ML) methods are integrated across all stages of the pipeline, enhancing data quality assessment, error correction, genotype calling, variant interpretation, kinship inference, and decision-support processes, ultimately improving accuracy, scalability, and robustness of forensic investigations.

Bioinformatic analysis in forensic genomics involves multiple steps, including sequence alignment, variant calling, quality control, and interpretation. These processes must be highly standardized and reproducible, particularly given the legal implications of forensic evidence. An overview of a typical AI-augmented forensic genomics pipeline is shown in Figure 2.

MPS-based forensic workflows require specialized pipelines capable of handling diverse marker types, including STRs, SNPs, microhaplotypes, and mitochondrial DNA. Unlike clinical genomics, where coverage depth and variant discovery are prioritized, forensic applications must also account for issues such as low-template DNA, allelic imbalance, and stochastic effects [5].

The convergence between forensic and clinical genomics is evident in the increasing adoption of standardized workflows and quality control metrics. Robust pipeline design—from library preparation to variant interpretation—is essential for ensuring data reliability, reproducibility, and analytical consistency across both forensic and clinical genomic applications [43,44]. This parallel evolution highlights the importance of cross-disciplinary knowledge transfer between clinical and forensic genomics.

### 6.2. Probabilistic Genotyping and Mixture Interpretation

One of the most challenging aspects of forensic DNA analysis is the interpretation of mixed DNA samples. Traditional approaches based on thresholding and manual interpretation are increasingly being replaced by probabilistic genotyping systems, which use statistical models to evaluate the likelihood of different contributor profiles.

Software tools such as STRmix and TrueAllele employ Bayesian frameworks to calculate likelihood ratios, incorporating factors such as allele drop-out, drop-in, and peak height variation [4]. These approaches have significantly improved the objectivity and reproducibility of mixture interpretation.

In forensic-statistical practice, probabilistic genotyping relies on the evaluation of alternative propositions, typically comparing prosecution and defense hypotheses regarding contributor composition. The evidential value of a DNA mixture is commonly expressed through likelihood-ratio (LR) frameworks, which quantify the relative probability of the observed genetic data under competing propositions [4,16,17]. Consequently, rigorous validation studies are essential to assess analytical sensitivity, reproducibility, stochastic effects, model robustness, and the impact of variables such as contributor number, contributor ratios, sequencing depth, and low-template DNA conditions. Sensitivity analyses and transparent reporting of model assumptions and uncertainty are particularly important when integrating MPS-derived sequence variation and emerging marker systems such as microhaplotypes into probabilistic interpretation workflows.

The integration of MPS data further enhances mixture analysis by providing sequence-level information and enabling the use of additional marker systems, such as microhaplotypes, which offer improved resolution in complex mixtures.

Although probabilistic genotyping systems are increasingly integrated with advanced computational approaches, Bayesian probabilistic frameworks should be conceptually distinguished from machine-learning-based predictive models and AI-assisted decision-support systems.

### 6.3. Machine Learning in Forensic Genomics

In parallel with probabilistic genotyping approaches, machine learning (ML) approaches are increasingly being applied across multiple domains of forensic genomics, including:

- Mixture deconvolution
- Phenotype prediction
- Ancestry inference
- Pattern recognition in genomic data

ML algorithms can identify complex, non-linear relationships within high-dimensional genomic datasets, making them particularly suitable for polygenic trait prediction and high-dimensional genomic analysis. For example, supervised learning models have been used to improve the accuracy of DNA phenotyping, while unsupervised approaches can assist in clustering and ancestry inference [9].

Despite their potential, ML models introduce challenges related to interpretability, validation, and generalizability. In forensic contexts, where transparency and explainability are critical, the use of “black-box” models remains a topic of ongoing debate.

A summary of the main applications of AI and machine learning in forensic genomics, along with their benefits and limitations, is presented in Table 5.

### 6.4. Integration of Multi-Source Data

One of the most significant contributions of AI and bioinformatics is the ability to integrate multiple layers of information, including:

- genetic markers (STRs, SNPs, microhaplotypes)
- genealogical data (FGG)
- phenotypic predictions (EVCs)
- demographic and geographical information

**Figure 3:**
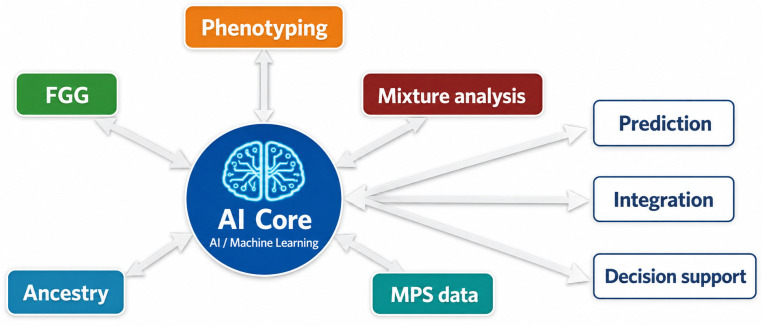
Role of AI in forensic genomics. Schematic representation of the central role of AI and machine learning in forensic genomics. The AI core functions as a transversal analytical layer that integrates multiple data streams, including MPS data, FGG, DNA phenotyping, ancestry inference, and mixture analysis. By combining heterogeneous genomic and contextual information, AI-driven approaches enable advanced analytical capabilities, including prediction of biological traits, data integration across multiple sources, and AI-assisted decision-support processes for forensic investigations. This framework highlights the transition from isolated analytical processes to a data-driven and intelligence-oriented forensic paradigm, where AI acts as a key enabler of comprehensive and scalable interpretation. The figure is intended as a conceptual representation of analytical integration rather than a quantitative model of evidential weighting or automated forensic decision-making.

**Figure 4:**
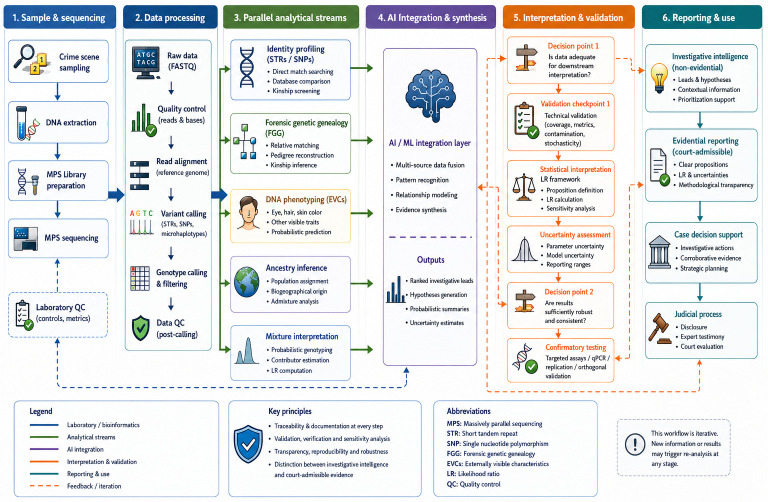
Integrated forensic genomics workflow with AI-assisted analytical integration, validation checkpoints, and evidential interpretation. A proposed operational workflow for integrated forensic genomics combining MPS, bioinformatic analysis, multi-source genomic inference, AI-assisted integration, and forensic-statistical interpretation. The workflow begins with crime scene sampling, DNA extraction, library preparation, and MPS sequencing, followed by quality control procedures, read alignment, variant calling, genotype filtering, and post-calling validation. Processed genomic data are subsequently analyzed across multiple complementary analytical streams, including identity profiling based on STRs and SNPs, FGG, FDP, ancestry inference, and probabilistic mixture interpretation. An AI/machine-learning integration layer combines heterogeneous genomic and contextual information to support pattern recognition, relationship modeling, probabilistic inference, and multi-source analytical synthesis. The workflow further incorporates explicit validation checkpoints, decision points, LR-based statistical interpretation, uncertainty assessment, and confirmatory testing procedures prior to evidential reporting and judicial use. Importantly, the model distinguishes exploratory forensic intelligence from court-admissible forensic evidence, emphasizing the need for methodological transparency, validation, reproducibility, and forensic-statistical rigor throughout the analytical process. Dashed arrows indicate iterative feedback loops, highlighting that new information or validation outcomes may trigger re-analysis at multiple stages of the workflow.

**Figure 5:**
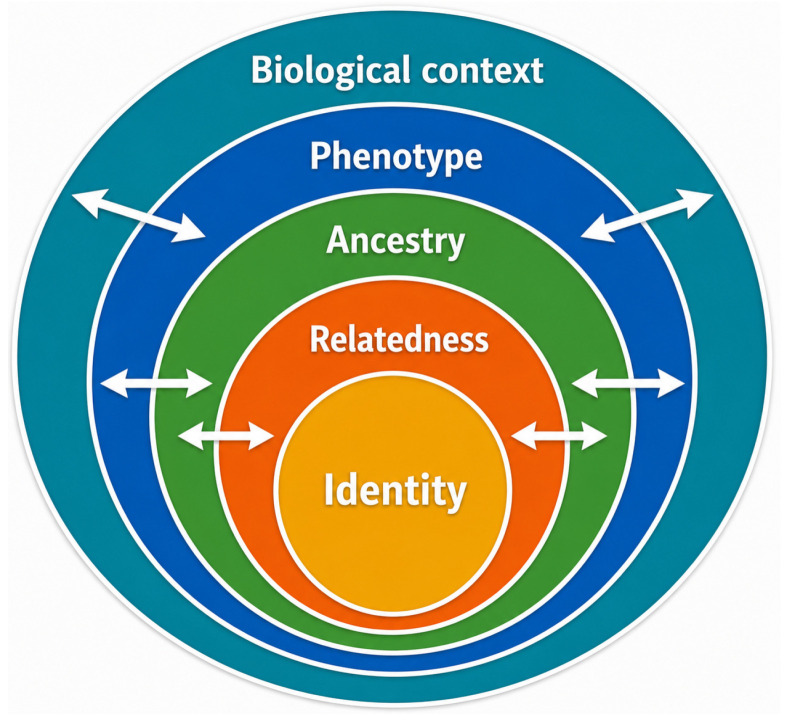
Multi-layered forensic information model. Schematic representation of the hierarchical and integrative structure of forensic genomic information. At the core, identity represents direct individual identification based on genetic profiles. Surrounding layers progressively expand the scope of inference, including relatedness (kinship and familial relationships), ancestry (biogeographical origin), phenotype (externally visible characteristics predicted from DNA), and biological context (age estimation, tissue origin, and epigenetic features). These layers should not be interpreted as representing equivalent evidential weight or hierarchical admissibility, but rather distinct categories of forensic information characterized by different analytical robustness, validation status, and forensic applicability. Bidirectional arrows indicate the dynamic interplay between layers, highlighting how different sources of genetic and epigenetic information can inform and refine each other within an integrated analytical framework. This model reflects the transition from single-layer identification toward multi-dimensional forensic intelligence, where diverse data types are combined to generate comprehensive investigative insights.

This multi-source integration enables a more comprehensive reconstruction of unknown individuals, moving beyond isolated analyses toward a systems-level approach. Such integration is central to the concept of forensic intelligence, where diverse data streams are combined to generate investigative leads (Figure 3). Figure 3, Figure 4 and Figure 5 illustrate complementary aspects of integrated forensic genomics, including AI-assisted analytical integration (Figure 3), operational forensic workflows combining multiple genomic approaches, validation checkpoints, and forensic-statistical interpretation (Figure 4), and the hierarchical organization of distinct categories of forensic inference (Figure 5). These figures are intended to provide conceptual, organizational, and operational perspectives on integrated forensic genomics rather than quantitative representations of evidential weighting or fully automated decision-making systems.

### 6.5. Challenges and Limitations

The implementation of AI and bioinformatics in forensic genomics presents several challenges:

- *Standardization*: lack of universally accepted pipelines and analytical standards
- *Validation*: need for rigorous validation of algorithms in forensic contexts
- *Interpretability*: difficulty in explaining complex models in court settings
- *Bias*: training datasets may introduce population-specific biases
- *Legal admissibility*: acceptance of AI-based evidence varies across jurisdictions

Addressing these challenges is essential to ensure the reliability and legal robustness of AI-driven forensic analyses.

### 6.6. Toward AI-Driven Forensic Intelligence

The integration of AI into forensic genomics marks a shift from data analysis to knowledge generation. Rather than simply processing genetic information, AI systems can identify patterns, generate hypotheses, and support decision-making processes.

This transformation aligns with the broader evolution of forensic science toward predictive and intelligence-driven frameworks. In this context, AI does not replace human expertise but augments it, enabling forensic scientists to interpret increasingly complex datasets with greater accuracy and efficiency.

## 7. Integration into Forensic Workflows

The convergence of MPS, FGG, DNA phenotyping, and AI-driven bioinformatics has led to the emergence of a new paradigm in forensic science: integrated forensic genomics. Rather than functioning as isolated analytical approaches, these technologies are increasingly combined within unified workflows that enable multi-dimensional interpretation of forensic evidence [7,8].

### 7.1. From Linear Pipelines to Integrated Systems

Traditional forensic DNA workflows follow a linear structure: sample collection, DNA extraction, amplification, STR profiling, and database comparison. While effective for direct identification, this model is limited when no database match is available.

In contrast, modern forensic genomics workflows are inherently multi-branching and iterative. Following DNA extraction and sequencing (typically via MPS), the resulting data can be processed simultaneously through multiple analytical streams:

- Identity analysis (STRs, SNPs)
- Kinship inference (FGG)
- Phenotype prediction (EVCs)
- Ancestry inference
- Mixture deconvolution and probabilistic genotyping

These parallel analyses are subsequently integrated to generate a comprehensive investigative profile of the unknown individual. A schematic overview of an integrated forensic genomics workflow is presented in Figure 4.

### 7.2. A Multi-Layered Information Framework

The integration of diverse genomic data types enables the construction of a multi-layered information framework, where each analytical component contributes a distinct level of insight:

- *Identity*: Direct matching to known profiles
- *Relatedness*: Kinship-based inference via FGG
- *Ancestry:* Biogeographical origin
- *Phenotype:* Externally visible characteristics
- *Biological context*: Age, tissue type (epigenetics)

This multi-dimensional framework reflects a shift from binary identification (match/no match) toward probabilistic and multi-dimensional inference. Importantly, these layers are not independent but interact dynamically, with information from one level informing and refining others (Figure 5).

### 7.3. Workflow Integration in Practice

In practical forensic scenarios, integrated workflows are particularly valuable in cases where traditional approaches fail.

A well-documented example is provided by the investigation leading to the identification of the ‘Golden State Killer’ in 2018, in which genome-wide SNP data generated from crime scene material were uploaded to a public genealogy database (GEDmatch), enabling the identification of distant relatives and the subsequent reconstruction of a family tree that led to the suspect [7,33,45]. While this case predates the full integration of AI-driven analytical tools, it illustrates the operational potential of combining MPS-based SNP profiling with genealogical inference.

More broadly, as a conceptual example, in a cold case with no STR database match:

- MPS enables SNP profiling from degraded DNA
- FGG identifies distant relatives through genealogy databases
- DNA phenotyping provides probabilistic predictions of physical appearance
- AI-based tools support the integration and prioritization of candidate investigative leads

When combined within appropriately validated forensic workflows, these complementary approaches may improve the generation and prioritization of investigative leads, particularly in complex cases involving degraded DNA or the absence of direct database matches. However, the analytical performance and forensic applicability of each component remain dependent on data quality, population representation, validation status, and legal or jurisdictional constraints.

Similarly, in missing persons and DVI, integrated genomics allows the simultaneous use of mtDNA, SNPs, and kinship inference to improve identification success rates.

### 7.4. Data Integration and Decision Support Systems

The integration of multi-source genomic data requires advanced computational frameworks capable of handling heterogeneous datasets. AI-driven decision support systems can synthesize outputs from different analytical modules, providing investigators with ranked hypotheses or probabilistic profiles. Recent studies have highlighted the growing role of machine learning in integrating heterogeneous forensic datasets and supporting decision-making processes [9].

However, in forensic contexts, ranked outputs and probabilistic prioritization systems require rigorous safeguards to minimize risks of confirmation bias, automation bias, and overinterpretation of exploratory results. Consequently, AI-assisted decision-support systems should be subject to transparent auditing procedures, calibration assessments, external validation, and continuous performance monitoring. Importantly, AI-generated hypotheses and probabilistic rankings should remain clearly separated from confirmatory forensic evidence and should be interpreted within validated forensic-statistical frameworks rather than as autonomous evidential conclusions.

Such systems represent a critical step toward operationalizing forensic intelligence, where data integration is not merely analytical but also decision-oriented. The development of standardized data formats and interoperable platforms will be essential for enabling widespread adoption.

An overview of the core components of integrated forensic genomics and their functional roles within modern forensic workflows is provided in Table 6.

### 7.5. Standardization and Validation Challenges

Despite its potential, the implementation of integrated forensic genomics workflows continues to face important challenges related to standardization, validation, and regulatory acceptance. While established forensic approaches such as STR profiling are supported by harmonized analytical frameworks, integrated workflows combining genomic, genealogical, phenotypic, epigenetic, and AI-assisted analytical components remain comparatively heterogeneous across laboratories and jurisdictions.

Importantly, integrated forensic genomics requires not only validation of individual analytical components, but also evaluation of the combined performance, reproducibility, uncertainty propagation, and interpretability of multi-source workflows across different forensic scenarios. These broader technical, analytical, ethical, and regulatory challenges are discussed in greater detail in Section 9.

### 7.6. Toward Predictive and Intelligence-Driven Forensic Science

The integration of genomic technologies fundamentally redefines the role of forensic genetics. Rather than serving solely as a tool for retrospective identification, forensic genomics is evolving into a proactive, intelligence-generating discipline.

This transformation is characterized by:

▪ the use of predictive models (phenotyping, ancestry)
▪ the incorporation of relational data (FGG)
▪ the application of AI for pattern recognition and data integration

Together, these elements support a shift toward forensic intelligence, where genomic data contribute to investigative strategies rather than simply confirming identity, enabling hypothesis generation and decision-making in complex forensic scenarios.

## 8. Ethical, Legal and Social Implications

The rapid evolution of forensic genomics, particularly with the integration of FGG, DNA phenotyping, and AI, has introduced a range of ethical, legal, and social implications (ELSI) that extend beyond traditional forensic practice. While these technologies significantly enhance investigative capabilities, they also challenge existing frameworks governing privacy, consent, data protection, and the admissibility of genetic evidence.

### 8.1. Genetic Privacy and Informed Consent

One of the most debated issues in modern forensic genomics concerns the use of genetic data obtained from individuals who have not explicitly consented to forensic applications. This is particularly relevant in the context of FGG, where law enforcement agencies may access public or semi-public genealogy databases to identify individuals through their relatives [32].

In such cases, individuals who have uploaded their genetic data for recreational or genealogical purposes may inadvertently expose their relatives to forensic investigation. This issue is particularly relevant in familial searching and FGG-based investigations, where individuals may become indirectly identifiable despite having never personally uploaded genetic data or consented to forensic use. Such scenarios raise complex questions regarding the ethical legitimacy and proportionality of indirect genetic surveillance through relatives. This phenomenon raises questions about the scope of informed consent and whether current consent models adequately reflect the potential uses of genetic data [37].

Moreover, the identifiability of individuals through distant relatives underscores the limitations of traditional notions of genetic privacy, suggesting that privacy in the genomic era is inherently collective rather than individual.

### 8.2. Data Protection and Regulatory Frameworks

Regulatory approaches to forensic genomics vary significantly across jurisdictions. In the United States, the use of FGG has been permitted under evolving guidelines, with increasing emphasis on transparency and case selection criteria. In contrast, European regulatory frameworks are shaped by the GDPR, national legislation, and law-enforcement-specific legal provisions governing the processing of sensitive genetic data. The forensic use of genetic information in Europe therefore depends not only on consent requirements, but also on legal basis, proportionality criteria, procedural safeguards, and jurisdiction-specific regulatory interpretations [45].

These regulatory differences create challenges for international collaboration and data sharing, particularly in cross-border investigations. Furthermore, the rapid pace of technological development often outpaces the establishment of clear legal frameworks, resulting in regulatory uncertainty.

Additional governance challenges concern the management, retention, auditing, and potential deletion of uploaded forensic SNP profiles used in genealogical investigations. Questions regarding who may access DTC databases, under which procedural safeguards, and for how long forensic genetic data may be retained remain incompletely harmonized across jurisdictions. The implementation of transparent audit trails, access logging, case eligibility criteria, and independent oversight mechanisms will therefore be essential for ensuring accountability and proportionality in forensic genomic investigations.

### 8.3. Ethical Concerns in DNA Phenotyping

DNA phenotyping raises additional ethical considerations, particularly regarding the potential for misuse or misinterpretation of predictive information. While FDP can provide valuable investigative leads, it also carries the risk of reinforcing stereotypes or contributing to racial profiling if not carefully contextualized [8].

The probabilistic nature of phenotypic predictions must be clearly communicated to avoid overinterpretation. For example, predictions of eye or skin color are not deterministic and may vary in accuracy depending on population background and genetic complexity. Importantly, probabilistic FDP and ancestry inference results must be communicated to investigators with appropriate contextualization and uncertainty estimates to minimize risks of overinterpretation, confirmation bias, or inappropriate narrowing of investigative focus.

Furthermore, the prediction of traits beyond externally visible characteristics—such as behavioral or health-related attributes—raises significant ethical concerns and is generally considered inappropriate for forensic use.

### 8.4. Bias and Representation in Genomic Databases

A critical issue in forensic genomics is the underrepresentation of certain populations in genetic databases. Most reference datasets used for SNP panels, phenotyping models, and genealogy databases are disproportionately composed of individuals of European ancestry [32].

This imbalance can lead to reduced accuracy in ancestry inference and phenotypic prediction for underrepresented populations, potentially introducing systemic bias into forensic investigations. Addressing this issue requires the development of more diverse and representative genomic datasets, as well as the implementation of bias-aware analytical methods.

### 8.5. AI, Transparency, and Accountability

The integration of AI and machine learning into forensic genomics introduces additional challenges related to transparency, accountability, and explainability. In legal contexts, the ability to explain how a result was obtained is critical for the admissibility of evidence.

However, many AI models—particularly deep learning approaches—operate as “black boxes,” making it difficult to trace the decision-making process. This lack of interpretability may undermine confidence in AI-derived evidence and complicate its use in court proceedings [9]. Moreover, AI-assisted analytical outputs used for investigative prioritization should remain clearly distinguished from confirmatory forensic evidence intended for courtroom admissibility. Maintaining this distinction is essential to prevent exploratory or probabilistic outputs from being interpreted as definitive evidential conclusions.

Ensuring that AI systems are transparent, validated, and auditable is essential for their responsible use in forensic science. The key ethical, legal, and social challenges associated with modern forensic genomics are summarized in Table 7.

### 8.6. Balancing Innovation and Responsibility

The integration of advanced genomic technologies into forensic workflows necessitates a careful balance between innovation and ethical responsibility. While these tools offer unprecedented capabilities for solving crimes and identifying individuals, their misuse or misinterpretation could have significant societal consequences.

Developing ethical guidelines, regulatory frameworks, and best practices is therefore essential to ensure that forensic genomics is applied in a manner that respects individual rights while supporting the goals of justice.

This balance will be particularly important as integrated forensic genomics increasingly combines probabilistic inference, AI-assisted analytical tools, and large-scale genomic databases within operational investigative workflows.

### 8.7. ELSI Within Integrated Forensic Genomics

Within the framework of integrated forensic genomics, ethical, legal, and social considerations are not peripheral but central to the responsible implementation of new technologies. As multiple data types are combined to generate increasingly detailed profiles, the potential impact on individuals and communities becomes more significant.

Consequently, ELSI considerations must be embedded within the design and application of forensic genomic workflows, ensuring that technological advancement is accompanied by ethical oversight and societal accountability. This includes ensuring that integrated forensic workflows maintain transparency regarding uncertainty, evidential limitations, data governance, and the distinction between forensic intelligence and court-admissible evidence.

## 9. Challenges and Future Directions

Despite the remarkable progress achieved in forensic genomics, several scientific, technical, and regulatory challenges remain to be addressed before fully integrated approaches can be routinely implemented in forensic practice. At the same time, emerging technologies and interdisciplinary advances are opening new directions that are likely to redefine the future of the field.

### 9.1. Technical and Analytical Challenges

One of the primary challenges in forensic genomics is the lack of standardized protocols for MPS-based analyses and multi-marker integration. While STR analysis benefits from decades of harmonization, emerging approaches involving SNPs, microhaplotypes, and epigenetic markers still lack universally accepted guidelines for data generation, interpretation, and reporting [5].

Data quality remains a critical issue, particularly in forensic contexts characterized by degraded DNA, low-template samples, and complex mixtures. Although MPS technologies have improved sensitivity, they also introduce additional sources of variability, including sequencing errors, allelic imbalance, and platform-specific biases.

Furthermore, the integration of heterogeneous data types—genetic, epigenetic, genealogical, and phenotypic—poses significant analytical challenges. Developing robust frameworks capable of combining these data in a coherent and reproducible manner remains an open problem.

### 9.2. Standardization and Validation

The implementation of integrated forensic genomics requires rigorous validation of both individual components and combined workflows. A major unresolved challenge is the absence of harmonized frameworks capable of validating not only individual analytical modules, but also the interaction, uncertainty propagation, reproducibility, and interpretability of integrated multi-source forensic workflows. In this context, validation extends beyond analytical accuracy and includes interoperability between platforms, robustness across heterogeneous forensic scenarios, and transparent reporting of probabilistic outputs. This includes the validation of emerging marker panels, benchmarking of bioinformatic pipelines, and inter-laboratory reproducibility studies.

The absence of standardized validation frameworks may limit the admissibility of evidence in legal settings and hinder the adoption of advanced methodologies.

### 9.3. Population Diversity and Bias

A major limitation of current forensic genomic tools is the underrepresentation of global population diversity in reference datasets. This affects multiple aspects of forensic analysis, including ancestry inference, DNA phenotyping, and FGG, leading to reduced accuracy and potential bias [41].

Future efforts must focus on expanding population databases to ensure equitable performance across diverse populations. Additionally, the development of bias-aware models and transparent reporting standards will be essential to mitigate these issues.

Addressing these limitations will be essential not only for improving predictive accuracy, but also for ensuring fairness, proportionality, and equitable forensic applicability across diverse population groups.

### 9.4. Ethical and Legal Barriers

Beyond current regulatory heterogeneity, a major future challenge will be the development of internationally interoperable governance frameworks capable of balancing forensic utility, transparency, proportionality, privacy protection, and judicial accountability. The increasing integration of genomic, genealogical, phenotypic, and AI-assisted analytical approaches raises complex questions regarding data access, oversight, auditability, and the distinction between forensic intelligence and court-admissible evidence. In particular, major concerns involve the use of genealogy databases, data protection and privacy safeguards, and informed consent or secondary use of genetic data.

These challenges require the development of clear regulatory frameworks that balance investigative needs with the protection of individual rights.

### 9.5. Emerging Technologies and Innovations

Several emerging technologies are expected to further reshape forensic genomics in the coming years:

▪ Portable sequencing platforms (e.g., nanopore sequencing) enabling on-site DNA analysis
▪ Single-cell genomics for resolving complex mixtures
▪ Long-read sequencing for improved haplotype resolution
▪ Multi-omics approaches integrating genomics, epigenomics, and transcriptomics

However, these technologies differ substantially in technological maturity, validation status, operational feasibility, and readiness for routine forensic implementation. Portable sequencing platforms and long-read sequencing approaches are increasingly approaching near-term forensic applicability in selected contexts, whereas single-cell genomics and comprehensive multi-omics integration currently remain more exploratory and research-oriented.

These innovations have the potential to enhance both the resolution and the scope of forensic analyses, particularly in challenging scenarios. Nevertheless, their broader implementation in routine forensic workflows will require rigorous validation, standardized analytical frameworks, cost-effectiveness assessments, and regulatory acceptance prior to judicial application.

### 9.6. Toward Predictive and Real-Time Forensic Genomics

Future forensic workflows are likely to become increasingly rapid, predictive, and data-driven. The integration of AI-assisted analytical pipelines may enable faster analysis and interpretation of genomic data, potentially supporting investigative lead generation in near real-time forensic contexts.

However, real-time forensic genomics remains insufficiently validated for routine operational implementation and would require rigorous contamination control, standardized validation procedures, secure data management, chain-of-custody safeguards, and confirmatory laboratory testing prior to judicial application.

### 9.7. The Future of Integrated Forensic Genomics

Ultimately, the future of Integrated Forensic Genomics will depend not only on technological innovation, but also on the development of transparent validation frameworks, interoperable analytical standards, explainable AI-assisted methodologies, population-aware reference resources, and internationally coherent governance structures. Achieving these goals will require sustained interdisciplinary collaboration among forensic scientists, bioinformaticians, statisticians, ethicists, legal experts, and regulatory institutions.

## 10. Conclusions

The field of forensic genetics is undergoing a profound transformation, driven by the convergence of massively parallel sequencing, forensic genetic genealogy, DNA phenotyping, and artificial intelligence. Together, these advances are revolutionizing forensic science from a discipline focused on individual identification to one capable of generating multi-dimensional forensic intelligence.

Traditional STR-based approaches, while still essential, are no longer sufficient to address the complexity of modern forensic challenges. The incorporation of SNPs, microhaplotypes, epigenetic markers, and genome-wide data has expanded the analytical toolkit, enabling the investigation of degraded samples, complex mixtures, and previously unsolvable cases.

At the same time, the integration of genealogical databases and predictive models has extended the scope of forensic genetics beyond direct matching, allowing for the reconstruction of biological relationships, physical traits, and population origins. These capabilities, further enhanced by AI-driven bioinformatics, support a shift toward data-driven and hypothesis-generating forensic workflows.

This review has proposed a conceptual framework of integrated forensic genomics, in which diverse technologies and data sources are combined within unified analytical pipelines. Within this framework, forensic genetics operates as a multi-layered system, integrating identity, relatedness, ancestry, phenotype, and biological context.

However, this transformation also introduces significant challenges, including issues of standardization, validation, bias, and ethical responsibility. Addressing these challenges will be essential to ensure the reliability, fairness, and public acceptance of advanced forensic methodologies.

Looking forward, the continued development of genomic technologies and computational tools is expected to further enhance the resolution, speed, and scope of forensic analyses. As these innovations advance, forensic genomics will increasingly function as an intelligence-driven discipline, contributing not only to the identification of individuals but also to the generation of investigative insights.

However, the routine implementation of integrated forensic genomics will require the establishment of rigorously validated MPS workflows, harmonized marker nomenclature and reporting standards, transparent and population-representative reference databases, and quantitative validation frameworks for forensic DNA phenotyping, ancestry inference, and forensic genetic genealogy. In parallel, the increasing integration of AI-assisted analytical systems will require explainable, auditable, and reproducible computational approaches that remain clearly separated from confirmatory forensic evidence intended for courtroom admissibility.

Ultimately, the future of integrated forensic genomics will depend not only on technological innovation but also on the ability of the forensic community to develop interoperable analytical standards, robust governance frameworks, and ethically responsible implementation strategies capable of balancing investigative utility, scientific rigor, and protection of individual rights in the genomic era.

## Figures and Tables

**Table 1:** Comparison of major forensic genetic marker systems.

Feature	STRs	SNPs	Microhaplotypes
Mutation rate	High	Low	Low
Amplicon size	Medium–long	Very short	Short
Suitability for degraded DNA	Moderate	High	High
Mixture resolution	Moderate-high	Moderate	High
Database compatibility	Extensive (CODIS-compatible)	Limited	Emerging
Information content	Length- and sequence-based	Sequence-based	Haplotype-based
MPS implementation	Established but analytically complex	Excellent	Excellent
Interpretation challenges	Stutter modeling, nomenclature harmonization	Population structure	Phasing and standardization

Abbreviations: STRs, short tandem repeats; SNPs, single nucleotide polymorphisms; CODIS, Combined DNA Index System; MPS, massively parallel sequencing.

**Table 2:** Emerging genetic and epigenetic marker systems and their forensic applications.

Marker Type	Key Features	Main Applications	Strengths	Limitations
SNPs	Single base variation	ID, ancestry, phenotyping	Short amplicons, stable	Lower discrimination than STRs
Microhaplotypes	Linked SNP clusters	Mixture analysis, ancestry	High informativeness	Limited standardization
InDels	Small insertions/deletions	ID (complementary)	Short fragments	Lower variability
mtDNA	Maternally inherited genome	Missing persons, degraded DNA	High copy number	Low individual specificity
Epigenetic markers	DNA methylation signatures	Age estimation, tissue identification	Functional and temporal inference	Environmental sensitivity and tissue specificity

Abbreviations: SNPs, single nucleotide polymorphisms; InDels, insertions and deletions; mtDNA, mitochondrial DNA; ID, identification; STRs, short tandem repeats.

**Table 3:** Comparison between traditional STR-based DNA profiling and FGG.

Feature	Traditional STR Profiling	Forensic Genetic Genealogy
Database type	Law enforcement (e.g., CODIS)	Public/DTC genealogy databases
Matching approach	Direct match	Kinship-based inference
Marker type	STRs	Genome-wide SNPs
Resolution	Individual identification	Identification via relatives
Applicability	Known offenders	Unknown individuals, cold cases
Ethical concerns	Limited	High (privacy, consent)

Abbreviations: STRs, short tandem repeats; FGG, forensic genetic genealogy; CODIS, combined DNA index system; DTC, direct-to-consumer; SNPs, single-nucleotide polymorphisms.

**Table 4:** Major phenotypic traits and representative predictive performance in forensic DNA phenotyping.

Trait	Genetic Architecture	Representative Predictive Metrics (a)	Key Genes/Markers	Forensic Utility
Eye color	Oligogenic	AUC often >0.90 for blue/brown prediction	*HERC2*, *OCA2*	Strong
Hair color	Oligogenic/polygenic	Accuracy commonly ~70–90% depending on color category	*MC1R*, *SLC45A2*	Strong
Skin pigmentation	Polygenic	AUC values vary substantially across populations and marker panels	*SLC24A5*, *TYR*	Moderate
Facial morphology	Highly polygenic	Robust and generalizable predictive metrics remain limited	Multiple loci	Emerging
Age estimation (epigenetic)	Epigenetic	Mean absolute error often ~3–5 years	CpG sites	Strong

(a) Representative predictive metrics are approximate and derived from published forensic DNA phenotyping studies using metrics such as area under the curve (AUC), classification accuracy, sensitivity, specificity, or mean absolute error, depending on the trait and predictive model. Reported performance may vary according to marker panels, analytical methods, population background, and validation datasets.

**Table 5:** Computational approaches in forensic genomics and their main applications.

Application Area	Computational Approach	Key Benefit	Limitations
Mixture interpretation	Bayesian probabilistic genotyping	Improved statistical interpretation and reproducibility	Model complexity and validation requirements
Phenotype prediction	Supervised machine learning	Enhanced predictive performance for complex traits	Population bias and overfitting
Ancestry inference	Clustering algorithms and machine learning approaches	Improved classification of population structure	Reference bias and population stratification
FGG data analysis	Network analysis and graph-based approaches	Reconstruction of kinship relationships	Dependence on database coverage
Multi-source integration	AI-assisted decision-support models	Interpretation of heterogeneous forensic data	Validation, explainability, and interoperability challenges

Abbreviations: FGG, Forensic Genetic Genealogy; AI, Artificial Intelligence .

**Table 6:** Core components of integrated forensic genomics.

Component	Data Type	Analytical Method	Output	Forensic Role
STR profiling	STRs	CE/MPS	DNA profile	Identification
SNP analysis	SNPs	MPS	Genotype data	Multi-purpose
FGG	SNP data and genealogy databases	Genealogical inference	Relatives	Lead generation
Phenotyping	SNPs	Predictive models	Physical traits	Intelligence
AI/Bioinformatics	Multi-source	ML/statistical models	Integrated profile	Decision support

Abbreviations: STR, Short Tandem Repeats; SNP, Single Nucleotide Polymorphisms; FGG, Forensic Genetic Genealogy; AI, Artificial Intelligence, CE, Capillary Electrophoresis; MPS, Massively Parallel Sequencing, ML, Machine Learning.

**Table 7:** Key ethical, legal, and social issues in forensic genomics.

Domain	Issue	Description	Implications
Privacy	Genetic identifiability	Identification via relatives	Collective privacy concerns
Consent	Secondary use of data	Use beyond original intent	Ethical/legal ambiguity
Regulation	Jurisdictional differences	GDPR vs. US frameworks	Limited harmonization
Governance	Auditability and data retention	Retention, deletion, and access monitoring of forensic SNP profiles	Accountability and proportionality concerns
Bias	Population underrepresentation	Reduced accuracy	Inequity in investigations
AI	Black-box models	Lack of interpretability	Legal admissibility issues

Abbreviations: AI, artificial intelligence; GDPR, general data protection regulation, US, United States; SNP, single nucleotide polymorphism.

## Data Availability

No new data were created or analyzed in this study.

## Abbreviations

The following abbreviations are used in this manuscript:

AI	Artificial Intelligence
AIMs	Ancestry-Informative Markers
CE	Capillary Electrophoresis
CODIS	Combined DNA Index System
DTC	Direct-to-Consumer
DVI	Disaster Victim Identification
ELSI	Ethical, Legal and Social Implications
EVCs	Externally Visible Characteristics
FDP	Forensic DNA Phenotyping
FGG	Forensic Genetic Genealogy
GDPR	General Data Protection Regulation
IBD	Identity by Descent
InDels	Insertions and Deletions
ML	Machine Learning
MPS	Massively Parallel Sequencing
mtDNA	Mitochondrial DNA
NGS	Next-Generation Sequencing
PCR	Polymerase Chain Reaction
QC	Quality Control
SNPs	Single Nucleotide Polymorphisms
STRs	Short Tandem Repeats
